# A Case of Severe Metabolic Acidosis due to Jardiance-Induced Euglycemic Diabetic Ketoacidosis

**DOI:** 10.7759/cureus.14580

**Published:** 2021-04-20

**Authors:** Nataliia Dyatlova, Yetunde B Omotosho, Robin Sherchan, Jishna Shrestha, Venkata Buddharaju

**Affiliations:** 1 Internal Medicine, The Chicago Medical School Internal Medicine Residency Program at Northwestern McHenry Hospital, McHenry, USA; 2 Internal Medicine, Northwestern Medicine McHenry Hospital, Rosalind Franklin University of Medicine and Science, McHenry, USA; 3 Nephrology, The Chicago Medical School Internal Medicine Residency Program at Northwestern McHenry Hospital, McHenry, USA

**Keywords:** jardiance, metabolic acidosis, edka

## Abstract

Metabolic acidosis is frequently encountered in the inpatient setting. It can occur due to either the accumulation of endogenous acids that consumes bicarbonate (high anion gap metabolic acidosis) or loss of bicarbonate from the gastrointestinal tract or the kidney.

Jardiance® (empagliflozin) (Boehringer Ingelheim Pharmaceuticals, Inc., Ridgefield, USA) is a sodium-glucose co-transporter 2 (SGLT2) inhibitor, which reduces renal tubular glucose reabsorption, thereby decreasing blood glucose level without stimulating insulin release. This class of drugs is known for reducing cardiovascular events and delay in the progression of chronic kidney disease in patients with type 2 diabetes mellitus (T2DM). However recent data has shown that SGLT2 inhibitors, particularly empagliflozin, carry the risk of inducing euglycemic diabetic ketoacidosis under certain circumstances such as acute illness, and decreased carbohydrate intake, decrease in dose, or discontinuation of insulin. We herein report a 23-year-old female with poorly controlled diabetes mellitus on empagliflozin, who presented with dyspnea and coronavirus disease SARS-CoV-2 (COVID-19) infection and found to have severe unexplained euglycemic metabolic acidosis, with elevated urine ketones.

## Introduction

Euglycemic diabetic ketoacidosis (EDKA) is a clinical syndrome occurring both in type 1 (T1DM) and type 2 (T2DM) diabetes mellitus characterized by euglycemia (blood glucose less than 250 mg/dL) in the presence of severe metabolic acidosis (arterial pH less than 7.3 and serum bicarbonate less than 18 mEq/L) and ketonemia [1]. Pathophysiology of EDKA explains carbohydrate deficit resulting in decreased serum insulin and excess counter-regulatory hormones like glucagon, epinephrine, and cortisol. This leads to increased lipolysis, increased free fatty acids, and ketogenesis [1-3]. However, the incidence of EDKA has grown with the introduction of sodium-glucose transporter 2 (SGLT2) inhibitors [4,5]. In the absence of typical characteristic hyperglycemia, there is always an initial confusion and risk of delay in treatment [1,6].

## Case presentation

We present a 23-year-old female with medical history of recent COVID-19 infection and uncontrolled diabetes mellitus currently on dulaglutide and empagliflozin who came to the emergency department with complaints of shortness of breath, dry cough, chills, malaise, diaphoresis. The patient denied any suicidal ideations or methanol/ethylene glycol intake. Physical exam revealed tachycardia and tachypnea. Laboratory data was remarkable for sodium bicarbonate (HCO3) of 11, anion gap 23, glucose 181, and hemoglobin A1C 9.5. Urinalysis was positive for glucose, protein, and ketones. She was admitted to the medical floor to manage metabolic acidosis and did not require treatment for COVID-19 infection. Oral hypoglycemic medications were held and the patient was started on sliding scale insulin. On day 2 of hospitalization, HCO3 decreased from 11 to 6. Arterial blood gas showed pH of 7.02, PCO2 16.7, PO2 67, and HCO3 4. Blood glucose increased to 224, and the anion gap increased to 24. Serum osmolarity was 295. Urine toxicology was negative. Nephrology service was consulted for further evaluation and management, Jardiance-induced euglycemic ketoacidosis was suspected. The patient was started on insulin and HCO3 infusion. On day 3, the anion gap decreased to 16, and insulin drip was discontinued and transitioned to subcutaneous insulin. The patient was continued on half-normal saline with 75 mEq of bicarbonate. She responded well to treatment with clinical improvement of symptoms. Subsequent laboratory data continued to show significant improvements with the closure of the anion gap. She was discharged on subcutaneous insulin injections, with instructions to discontinue Jardiance and follow up with endocrinology.

Chart review demonstrated that patient was supposed to be on insulin therapy and was not following up with the endocrinologist and ran out of insulin before this admission for DKA. COVID-19 infection, though, likely additionally triggered the development of DKA, euglycemic in the settings of Jardiance.

## Discussion

Diabetic ketoacidosis (DKA) and hyperglycemia hyperosmolar state (HHS) are the most severe acute metabolic emergencies in type 1 and type 2 diabetes. Ketosis results from restriction of carbohydrate usage with increased reliance on fat oxidation for energy production. DKA presents with marked hyperglycemia (>250 mg/dl, typically 350-800 mg/dl), profuse glycosuria, and hyperketonemia [5]. Euglycemic DKA (EDKA) is a clinical syndrome occurring in both type 1 and type 2 diabetes mellitus characterized by euglycemia (blood glucose <250 mg/dl) in the presence of severe metabolic acidosis (arterial pH <7.3 and serum bicarbonate <18 mEq/L) and ketonemia [1]. The incidence of EDKA has grown with the introduction of sodium-glucose transporter 2 (SGLT-2) inhibitors [7]. About 2.6% of DKA admissions are euglycemic [2]. DKA associated with SGLT-2 inhibitors ranges between 0.16-0.76 events per 1000 patient-years in patients with type 2 diabetes [4]. The pathogenesis of SGLT-2 inhibitor-associated EDKA reveals that by competitive inhibition of SGLT-2 at the proximal convoluted tubule, SGLT-2 inhibitors block the reabsorption of 30-50% of filtered glucose from the primary urine [8]. The hypoglycemic effect of this “carbohydrate deficit” is only partly offset by the increased endogenous glucose production via gluconeogenesis and decrease glucose disposal. The lower blood glucose causes a decrease in circulating insulin and an increase in glucagon concentration. Decreased reabsorption of ketones also contributes to ketonemia [1-3]. 

**Figure 1:**
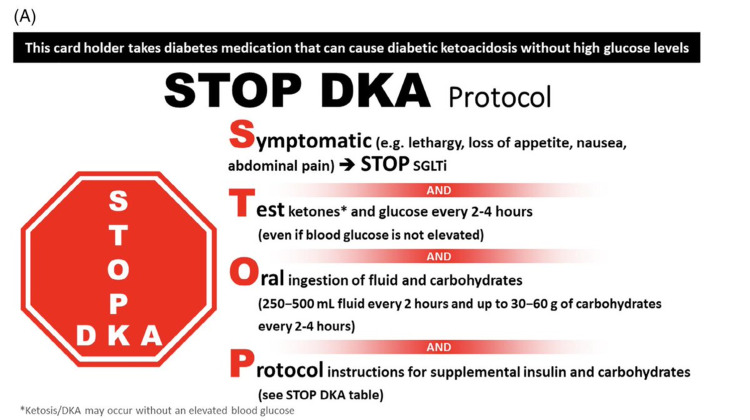
Stop DKA Protocol

**Figure 2:**
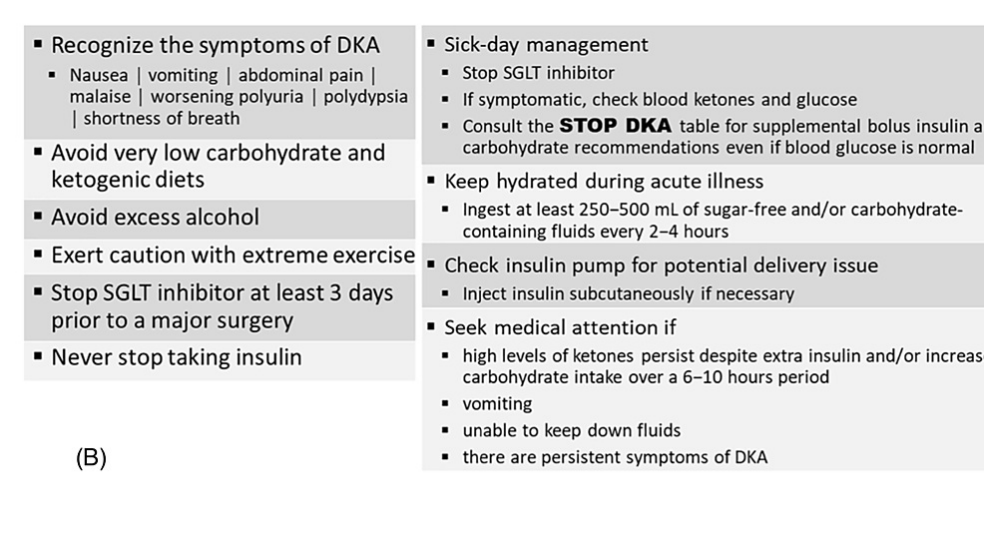
Stop DKA Table

SGLT-2 inhibitors are known for significant renal and cardiovascular benefits, such as lowering blood pressure, impact on heart failure, kidney protection, and mortality, demonstrated in multiple studies [9-13]. This class of diabetic medications became popular, especially among people with chronic kidney disease (CKD) who have limited oral diabetic medication options [14]. Euglycemic DKA is a known side effect associated with starvation, a strict diet with decreased carbohydrate intake, missed or decreased insulin doses, and acute illness. It is crucial to emphasize that SGLT-2 inhibitors are not approved for type 1 diabetes due to a lack of data regarding glycemic efficacy during long-term use. Also, it was observed that this class of medications dramatically increases the risk of DKA in type 1 diabetics [15]. SGLT2 inhibitors are an effective, well-tolerated, and very beneficiary class of antidiabetic medications, but the more we use them, the more we observe EDKA as a side effect. Given how reasonably popular this class became lately, it is crucial to understand that SGLT2 inhibitors are not harmless and carry only great benefit, but they should be used with caution, awareness, and extensive patients counseling [7]. To minimize the risk of DKA and still gain the multiple benefits, "STOP DKA Protocol" was developed - an easily accessible and practical tool that provides a risk mitigation strategy for reducing DKA in patients with diabetes being treated with SGLT inhibitors [16] (Figures 1,2). 

## Conclusions

Euglycemic DKA is a diagnostic challenge for physicians due to the variety of etiologies and normal blood glucose levels, often resulting in delayed diagnosis. There are many causes of EDKA in patients with diabetes. However the growing incidence of EDKA and metabolic acidosis induced by SGLT2 inhibitors warrants awareness and caution amongst caregivers. Patients with type 1 or type 2 diabetes who experienced nausea, vomiting, malaise, or develop metabolic acidosis in the setting of SGLT-2 inhibitor therapy should be promptly evaluated for the presence of urine and/or serum ketones.
